# The evolution and mechanism of GPCR proton sensing

**DOI:** 10.1074/jbc.RA120.016352

**Published:** 2020-12-13

**Authors:** Jacob B. Rowe, Nicholas J. Kapolka, Geoffrey J. Taghon, William M. Morgan, Daniel G. Isom

**Affiliations:** 1 The Department of Molecular and Cellular Pharmacology, University of Miami Miller School of Medicine, Miami, Florida, USA; 2 The Department of Tumor Biology, University of Miami Sylvester Comprehensive Cancer Center, Miami, Florida, USA; 3 The Institute for Data Science Computing, University of Miami, Coral Gables, Florida, USA

**Keywords:** G protein–coupled receptor, proton sensing, proton, sodium, coincidence detection, allosteric modulator, evolution, BRET, bioluminescence resonance energy transfer, CNA, consensus network analysis, DCyFIR, Dynamic Cyan Induction by Functional Integrated Receptors, DVP, deep variant profiling, eHis, extracellular His, GPCR, G protein–coupled receptor, mGp, mini G protein, NAM, negative allosteric modulator, NES, nuclear export sequence, PDB, Protein Data Bank, rSites, reported sites, SCD, synthetic complete dextrose

## Abstract

Of the 800 G protein–coupled receptors (GPCRs) in humans, only three (GPR4, GPR65, and GPR68) regulate signaling in acidified microenvironments by sensing protons (H^+^). How these receptors have uniquely obtained this ability is unknown. Here, we show these receptors evolved the capability to sense H^+^ signals by acquiring buried acidic residues. Using our informatics platform pHinder, we identified a triad of buried acidic residues shared by all three receptors, a feature distinct from all other human GPCRs. Phylogenetic analysis shows the triad emerged in GPR65, the immediate ancestor of GPR4 and GPR68. To understand the evolutionary and mechanistic importance of these triad residues, we developed deep variant profiling, a yeast-based technology that utilizes high-throughput CRISPR to build and profile large libraries of GPCR variants. Using deep variant profiling and GPCR assays in HEK293 cells, we assessed the pH-sensing contributions of each triad residue in all three receptors. As predicted by our calculations, most triad mutations had profound effects consistent with direct regulation of receptor pH sensing. In addition, we found that an allosteric modulator of many class A GPCRs, Na^+^, synergistically regulated pH sensing by maintaining the p*K*_a_ values of triad residues within the physiologically relevant pH range. As such, we show that all three receptors function as coincidence detectors of H^+^ and Na^+^. Taken together, these findings elucidate the molecular evolution and long-sought mechanism of GPR4, GPR65, and GPR68 pH sensing and provide pH-insensitive variants that should be valuable for assessing the therapeutic potential and (patho)physiological importance of GPCR pH sensing.

Since their discovery in 2003 (1), G protein–coupled receptor (GPCR) proton sensors have been implicated in a variety of biological processes and disorders (2, 3, 4, 5, 6, 7, 8, 9, 10), including ischemia (11, 12, 13), inflammation (14, 15, 16), and signaling in tumor microenvironments (17, 18, 19, 20, 21, 22, 23, 24, 25, 26). The acidotic cues generated by these processes are known to stimulate GPCR signaling thought to contribute to a variety of cellular responses, such as fibroblast proliferation in cancer and insulin secretion (19, 27). However, despite these physiological insights, the pH-sensing receptors GPR4, GPR68 (OGR1), and GPR65 (TDAG8) maintain their status as understudied and “pharmacologically dark” receptors (28, 29). One approach for illuminating the biological importance of these receptors is to understand how they evolved the ability to sense protons. This insight would provide both a mechanistic explanation for GPCR pH sensing and enable the engineering of functional receptor variants insensitive to physiological pH changes, which has yet to be achieved. Such variants would finally enable a true assessment of the effects and physiological importance of GPCR pH sensing in a variety of *in vitro* and *in vivo* model systems.

Elucidating the mechanisms of macromolecular pH sensing remains a formidable computational and experimental challenge. As a result, there are relatively few known pH-sensing proteins (pH sensors) and even fewer examples of discrete pH-sensing mechanisms. This lack of insight has prevented us from recognizing the broader implications for pH sensing throughout biology and disease. Like well-known pH sensors, such as hemoglobin (30), GPCR pH sensors can only detect protons using five amino acids: aspartic acid (Asp, D), glutamic acid (Glu, E), histidine (His, H), arginine (Arg, R), and lysine (Lys, K). Most proteins are insensitive to physiological pH changes because their ionizable residues are constantly charged between pH 5 and 7.4. In contrast, pH sensors contain specialized ionizable residues that (un)charge in response to physiological pH changes to regulate protein structure, function, and biology. In the case of GPCR pH sensors, such residues detect and transduce extracellular proton signals to activate intracellular signaling pathways and elicit appropriate, context-dependent cellular responses.

Although pH sensing by His residues is widely recognized (31, 32), the important pH-sensing roles of other ionizable amino acids are underappreciated (33, 34, 35). His is conceptually associated with pH sensing because its p*K*_a_ value of 6.6 ± 1.0 is in the physiological pH range (36). However, the more extreme p*K*_a_ values of acidic Asp and Glu residues (typically <4.5) (36), and basic Arg and Lys residues (typically >10) (36), can be shifted into the physiological pH range when buried in the protein interior (33, 34, 35). As such, buried Asp, Glu, Arg, and Lys residues can make important contributions to pH-sensing mechanisms and serve as important predictors in the search for proteins that couple changes in pH to the regulation of biological function. Previously, we have shown that GPCRs often contain buried ionizable residues (37); however, it is unclear whether such residues are important for the physiological control of GPCR signaling by pH signals.

In this study, we show that 3 human GPCRs evolved the ability to sense pH by acquiring buried acidic residues that emerged in GPR65 and were later inherited by GPR4 and GPR68. Before this finding, pH sensing by these receptors was thought to originate solely from extracellular His (eHis) residues (1, 22, 38, 39). However, most of these eHis residues appeared in GPR4 and GPR68 after GPR65 evolved the ability to sense protons. This advance in our understanding of GPCR pH sensing was made possible by our informatics platform, known as pHinder (40), which we created for identifying pH-sensing structural features that are often overlooked. In the case of GPR4, GPR65, and GPR68, our pHinder calculations identified a triad of buried acidic residues that have escaped the attention of the field for almost 2 decades. Here, we used our new yeast-based technology, deep variant profiling (DVP), along with GPCR assays in HEK293 cells and phylogenetic analyses to confirm our computational prediction that these residues are responsible for the molecular evolution and mechanism of GPCR pH sensing.

## Results and discussion

### GPR4, GPR65, and GPR68 share a unique triad of buried acidic residues

pHinder is an informatics program that uses computational geometry to identify structural features, such as buried ionizable residues, that are predictive of pH sensing (40). In the pHinder algorithm (see Fig. S1*A*), Asp, Glu, His, Lys, and Arg residues are triangulated to calculate a topological network of ionizable residues in a protein. A molecular surface is then calculated and used to identify buried network nodes that reside below the surface. In this work, we used such calculations to identify a distinctive network of buried acidic residues in homology models of GPR4, GPR65, and GPR68 pH sensors. Drawing from our extensive experience studying such residues in other proteins, we hypothesized that the neutral (protonated) ensemble of these buried acidic residues was directly responsible for acid-activated GPCR signaling, and thus pH sensing.

**Figure 1:**
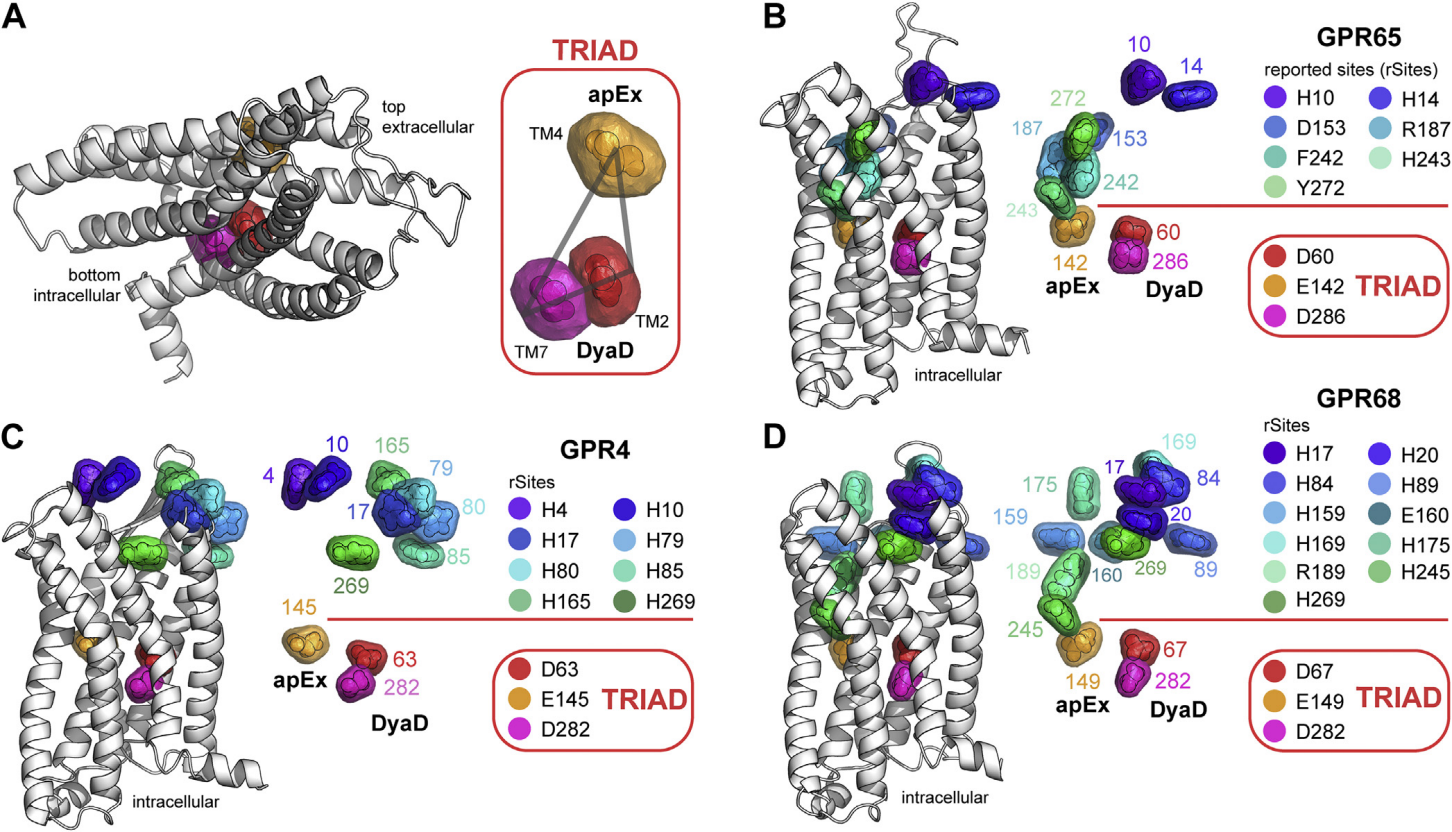
**GPR4, GPR65, and GPR68 share a triad of buried acidic residues.***A*, homology model of GPR65 showing the triad of buried acidic residues that originate from transmembrane helix 4 (TM4) (a Glu residue we call the apEx site) and TM2 and TM7 (a pair of Asp residues we call the DyaD site). *B*–*D*, Homology models of GPR65 (*B*), GPR4 (*C*), and GPR68 (*D*) showing the location of the buried acidic triad and 26 reported sites (rSites) of all GPR4, GPR65, and GPR68 mutations available in the literature. An illustration of the pHinder algorithm for identifying networks of buried ionizable residues is available in Fig. S1.

As shown in Figure 1, all 3 GPCR pH sensors share a triad of buried acidic residues not observed in 416 other human GPCR structures (Fig. S1*B*). As illustrated in Figure 1*A*, we refer to 2 of the 3 acidic residues as the DyaD site because they comprise a pair of closely spaced Asp residues originating from transmembrane helices 2 and 7. Notably, Na^+^ ions observed in several GPCR structures map to the vicinity of the DyaD site (Fig. S1*C*), suggesting that Na^+^ may have the potential to modulate GPCR pH sensing. The third acidic residue of the triad, which we call the apEx residue, is a Glu that originates from transmembrane helix 4 and is located ∼16 Å from the DyaD site. In all 3 GPCRs (Fig. 1, *B*–*D*), the buried acidic triad is positioned approximately horizontal to the plasma membrane near the midpoint of each receptor. In addition to the buried acidic triads, Figure 1, *B*–*D* shows the 26 residue positions that have been investigated in prior studies of GPCR pH sensing (1, 22, 38, 39). We collectively refer to these positions as reported sites (rSites), which correspond to locations in and near the orthosteric ligand-binding sites of other class A receptors.

Our hypothesis that these buried acidic triads are responsible for GPCR pH sensing predicts several possible outcomes. Because protonation of the triad ensemble at low pH should activate all 3 receptors, isosteric Asn and Gln mutations that mimic the neutral forms of Asp and Glu side chains should result in receptor variants that retain function yet signal independent of extracellular pH below ∼7.4. This outcome would also confirm that triad residues have dramatically upshifted p*K*_a_ values. Furthermore, favorable electrostatic interactions between DyaD residues and Na^+^, a known negative allosteric modulator (NAM) of GPCR signaling (41), should reduce the magnitude of DyaD site p*K*_a_ shifts to maintain GPCR pH sensing within the physiologically relevant pH range. This outcome would also confirm that the DyaD residues are directly involved in the pH-sensing mechanism. Finally, because buried ionizable residues are usually destabilizing (33, 34, 35), their removal by Asn and Gln mutations should increase GPCR stability to maintain or enhance signal efficacy. As will follow, we successfully confirmed this set of predicted outcomes using both a new method we developed for high-throughput mutational profiling in yeast and *in vitro* assays in HEK293 cells.

### DVP of GPR4, GPR65, and GPR68

**Figure 2:**
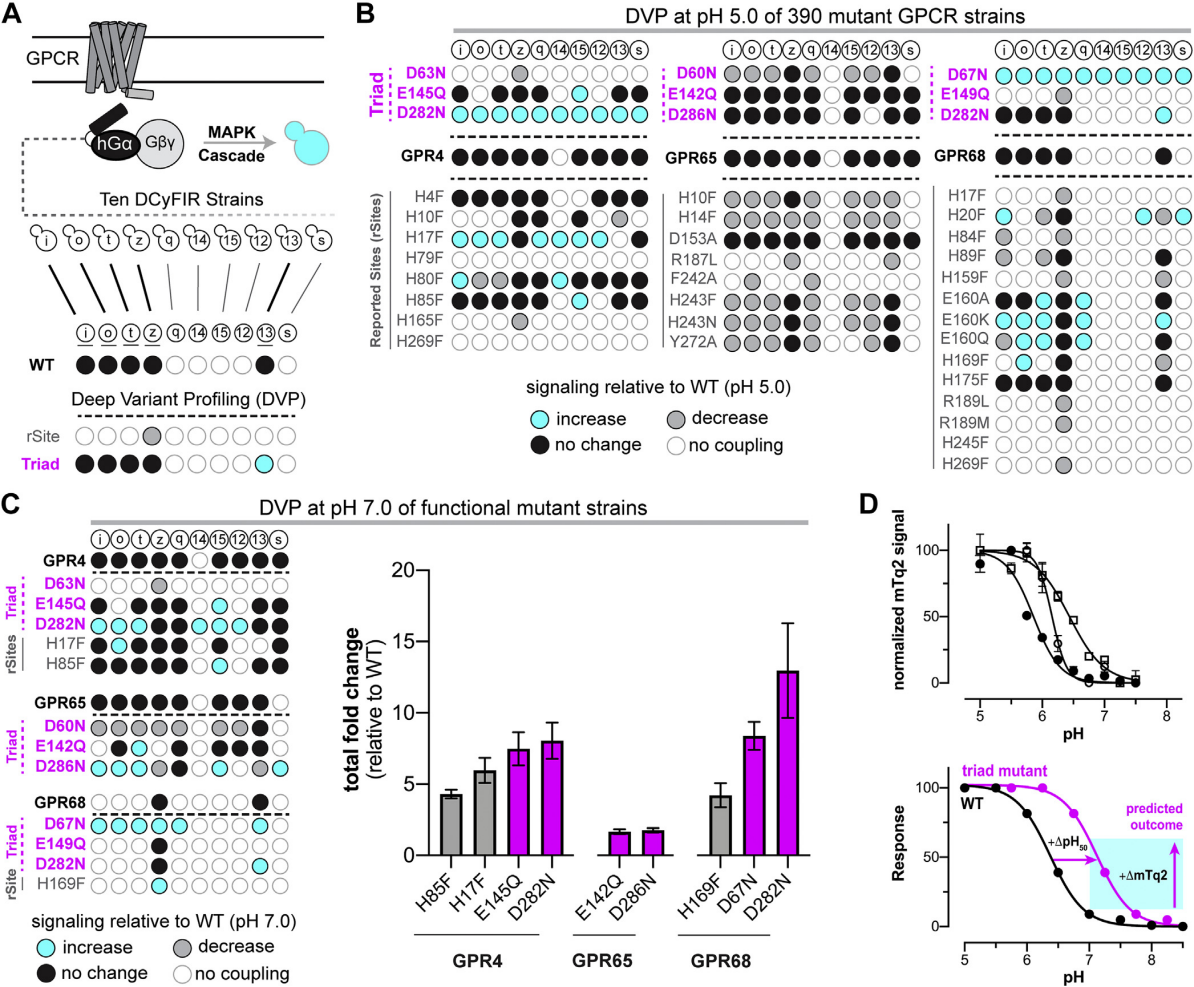
**Deep variant profiling of GPR4, GPR65, and GPR68 at pH 5.0 and 7.0.***A*, the DCyFIR strain platform and DVP technology for rapidly screening human GPCRs and their mutants in yeast (42, 43). Each DCyFIR strain consists of a human GPCR and a humanized Gα chimera (hGα). GPCR activation and subsequent hGα coupling result in expression of the fluorescent transcriptional reporter, mTurquoise2. DVP is used to interrogate the functional significance of GPCR residues. Data from GPR68 (WT), GPR68–H17F (rSite), and GPR68–D282N (triad) were used here to demonstrate the approach. *B*, DVP of 390 DCyFIR strains at pH 5.0. GPCR signaling in each mutant strain was quantified relative to the WT by calculating the log_2_ fold change (FC) in mTq2 fluorescence (*n* = 2). Results were scored as an increase in signaling (*cyan*; log_2_ FC > 0.5), no change in signaling (*black*; log_2_ FC ± 0.5), decrease in signaling (*gray*; log_2_ FC < −0.5), and no signaling or Gα coupling (*white*). *C*, *left*, DVP of the 195 functional DCyFIR-strain mutants at pH 7.0. Results are shown for the 9 triad sites and the only 3 His to Phe rSite mutations that exhibited increased signaling at pH 7.0. Coloring is based on log_2_ FC in mTq2 fluorescence (*n* = 2) and scored as an increase in signaling (*cyan*; log_2_ FC > 2.0 for GPR4 and GPR68; log_2_ FC > 0.5 for GPR65), no change in signaling (*black*; log_2_ FC ± 2.0 for GPR4 and GPR68; log_2_ FC ± 0.5 for GPR65), decrease in signaling (*gray*; log_2_ FC < −2.0 for GPR4 and GPR68; log_2_ FC < −0.5 for GPR65), or no detectable signaling or Gα coupling (*white*). *C*, *right*, Summed log_2_ FC values for the DCyFIR strains of each mutant in the left panel. Error bars represent SD (*n* = 2–12). *D*, *top*, pH profiles of WT GPR4 (*open circles*), GPR65 (*open squares*), and GPR68 (*closed circles*) measured using the Gα_i_ DCyFIR strain. Data are the mean ± SD (*n* = 4). *D*, *bottom*, Based on our predictions, mutation of Asp and Glu triad residues to permanently neutral Asn or Gln side chains should cause right-shifted midpoints of proton activation (pH_50_ values) and greater signaling as indicated by increased mTq2 fluorescence at and above pH 7.0. Primary data, numerical details, and experimental errors for the data in panels *B* and *C* can be found in Figures S2 and S3. DCyFIR, Dynamic Cyan Induction by Functional Integrated Receptors; DVP, deep variant profiling.

Recently, we developed Dynamic Cyan Induction by Functional Integrated Receptors (DCyFIR), a yeast-based platform for rapidly profiling ligand responses by human GPCRs (42, 43). In the DCyFIR platform, a genome-integrated human GPCR couples to a human/yeast C-terminal Gα chimera to drive the expression of a cyan transcriptional reporter (mTurquoise2) (Fig. 2*A*). DCyFIR compliments more traditional *in vitro* GPCR assays in several unique ways. Because yeast have only one GPCR pathway, grow rapidly, are pH tolerant, and do not require stringent cell culture techniques, the DCyFIR system is ideal for high-throughput experimental formats. In addition, our ability to install and barcode yeast strains with human GPCRs using high-throughput CRISPR engineering adds to the utility and versatility of DCyFIR profiling (42, 43). Finally, the DCyFIR strain collection enables us to independently interrogate individual GPCR–Gα coupling interactions for all 10 possible GPCR–Gα coupling combinations (Fig. 2*A*).

Here, we introduce a new DCyFIR-based method called DVP. Using DVP, we can rapidly build DCyFIR strain libraries that assess the effects of GPCR SNPs, test mutations in the process of developing designer GPCRs and biosensors, and, as in this study, evaluate residues computationally predicted to regulate GPCR structure–function relationships. As such, the insights gained from higher throughput DVP studies can be used to prioritize and inform lower throughput studies in mammalian cell models. Here, we used DVP to characterize each component of the buried acidic triads in GPR4, GPR65, and GPR68 (9 total mutations), along with all 26 rSites (Fig. 2*B*). Because we test all 10 possible GPCR–Gα coupling interactions in the DCyFIR system (Fig. 2*A*), our DVP experiments required us to build a library of 420 DCyFIR strains using our high-throughput CRISPR pipeline.

DVP at pH 5.0 showed that 36 of 39 mutant receptors (9/11 GPR4, 11/11 GPR65, and 16/17 GPR68) were functional in 195 DCyFIR strains (58/110 GPR4, 77/110 GPR65, and 60/170 GPR68 strains) (Fig. 2*B*). Consistent with prior work (1, 39), the 3 loss-of-function mutants identified by DVP (GPR4–H79F, GPR4–H269F, and GPR68–H245F) were rSite mutations already shown to have deleterious effects. As predicted, most triad mutants (7 of 9) signaled as well or better than WT in one or more DCyFIR strains (Fig. 2*B* and Fig. S2). The two exceptions were the DyaD mutant GPR4–D63N and apEx mutant GPR68–E149Q, the latter of which is complicated by weak WT GPR68 signaling, an observation consistent with our prior finding and explanation of weak GPR68 signaling in the yeast system (43). Furthermore, we found most triad mutants maintained WT Gα coupling patterns and in two cases gained newly detectable Gα coupling interactions: DyaD mutants GPR4–D282N and GPR68–D67N. In contrast, and consistent with prior studies (1, 22, 38, 39), we found most rSite mutants exhibited a reduction or loss of signaling, with limited instances of new and weakly detectable signaling: GPR4–H17F, GPR4–H80F, GPR68–H20F, and GPR68–E160Q/A/K. As shown in Figure 2*C* and Figure S3, additional DVP at pH 7.0 identified a total of 11 mutants, including 6 of 9 triad mutants that outperformed the WT. Based on the pH profiles of all three WT receptors (Fig. 2*D*, top), this finding was consistent with the behavior we predicted for triad mutations (Fig. 2*D*, bottom) and confirmed that rSite residues collectively make minimal contributions to GPR4, GPR65, and GPR68 pH sensing.

### *In vitro* validation of triad residues responsible for GPCR pH sensing in HEK cells

**Figure 3:**
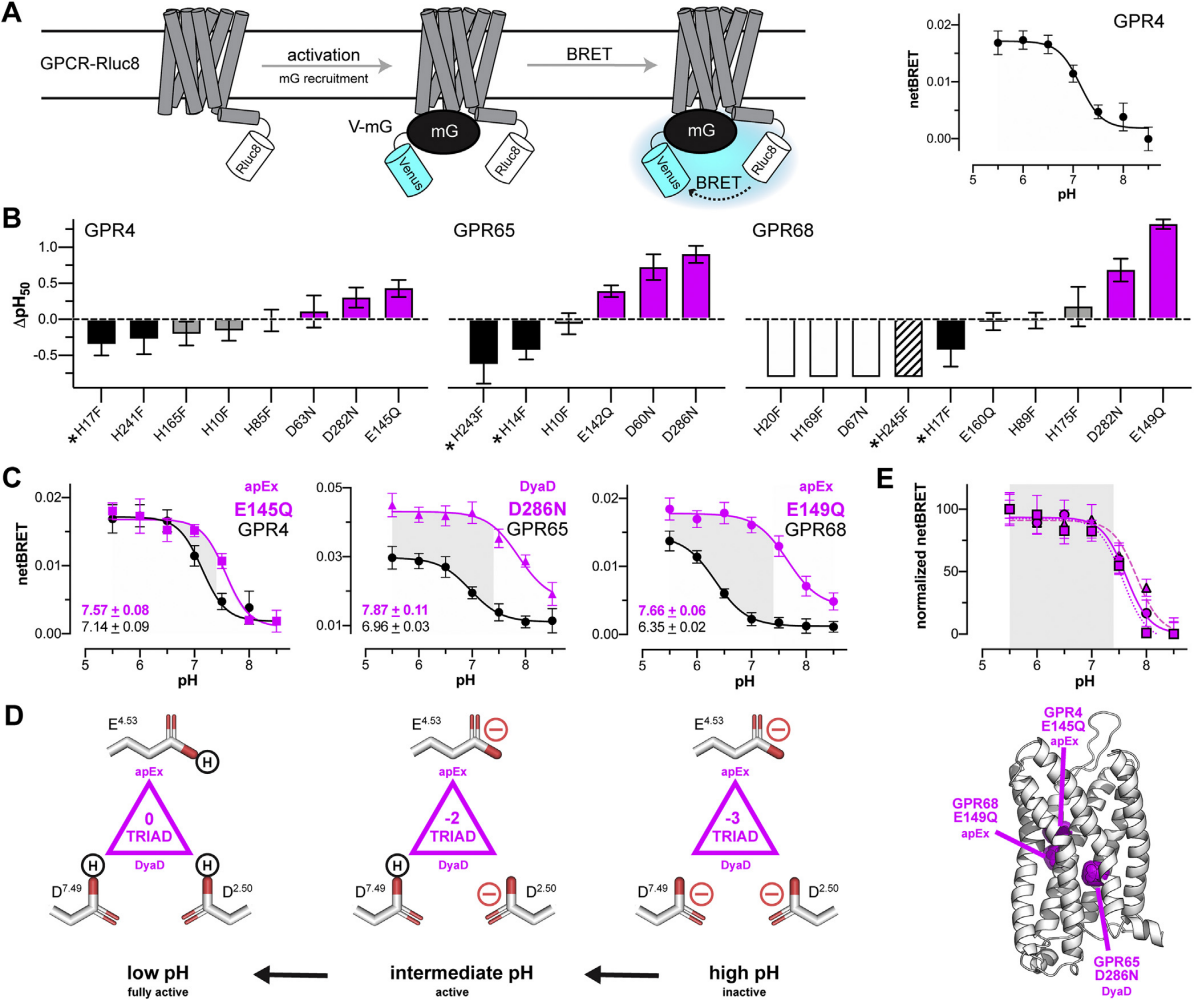
**Validating the mechanism of GPR4, GPR65, and GPR68 proton sensing in HEK293 cells.***A*, the BRET-based mini G protein (mGp) assay used in HEK293 cells. Coupling of a Venus-tagged mGp (V-mG) to a luciferase-tagged GPCR (GPCR–Rluc8) produces a BRET signal (*left*) that can be used as a direct readout of GPCR pH activation profiles, as shown for the representative pH profile of WT GPR4 (*right*). *B*, relative pH_50_ changes (ΔpH_50_) of 24 GPR4, GPR65, and GPR68 triad (*pink*) and rSite (*gray*) variants and His to Phe variants at sites shared by all 3 GPCRs (*black*, *solid*, or *striped*). Variants indicated by an *asterisk* correspond to both rSite and conserved His sites. *White bars* (*solid* or *striped*) indicate nonfunctional variants (*e.g.*, GPR68–D67N and –H245F) or variants with pH_50_ values below the measured pH range (*e.g.*, GPR68–H20F and –H169F). Data are the mean ± SEM of the net BRET signal (netBRET) (*n* = 3–11). *C*, pH profiles of select triad variants (*pink*) that exhibited upshifted pH_50_ values and nullified pH sensing within the physiologic pH range (*gray shading*). *Black curves* correspond to the pH profiles of WT receptors. Inset values are the pH_50_ values (mean ± SEM) for each triad variant and its corresponding WT receptor. *D*, interpretive model of triad (de)protonation and GPCR activation as a function of pH. The net charge and sequence of titration steps is only intended to illustrate the combination of (de)protonation events that underlie the pH-sensing mechanism. *E*, overlaid pH profiles and structural mapping of pH-insensitive variants shown in panel *C* (GPR4–E145Q, *squares*, *dotted line*; GPR65–D286N, *triangles*, *dashed line*; and GPR68–E149Q, *circles*, *solid line*). Data were normalized by adjusting the smallest and largest netBRET values for each variant to 0 and 100, respectively. *Gray shading* indicates the physiologically relevant range for GPCR proton sensing. Data shown in panels *A*–*C* are the mean ± SEM (*n* = 5–9). Mini G protein Venus–mGsi (V-mGsi) was used for all data in panels *A*–*C* to be consistent with our yeast titration data in Figure 2*D*. Thirty three additional pH profiles related to panel *B* can be found in Figure S4, *B*–*D*. BRET, bioluminescence resonance energy transfer; GPCR, G protein–coupled receptor.

Guided by the outcome of our high-throughput DVP experiments, we next performed lower throughput pH titrations using a bioluminescence resonance energy transfer (BRET) mini G protein (mGp) assay in HEK293 cells (44). As illustrated in Figure 3*A* and Figure S4*A*, we chose the mGp assay because it enabled us to directly quantify the effects of pH on GPCR signaling using luciferase-tagged receptors (BRET donors) and mGps tagged with the fluorescent protein Venus (BRET acceptors). Using this approach, we titrated the pH responses of the 9 triad mutants, 14 rSite mutant controls, and the only 2 His residues conserved in all 3 receptors. As predicted, 7 of 9 triad mutants had right-shifted midpoints of proton activation (pH_50_ values) and increased signaling at pH 7.4 relative to WT (Fig. 3*B* and Fig. S4), an outcome never achieved by His mutations (1, 22, 38, 39). As shown by the representative pH profiles in Figure 3*C*, the mutation of both DyaD (GPR65–D286N) and apEx (GPR4–E145Q and GPR68–E149Q) sites results in pH sensor variants that have signal efficacy equivalent to or better than WT and right-shifted pH_50_ values (Fig. 3, *B*–*C*). As expected, the relative pH insensitivity indicated by the weak signaling of the GPR68–E149Q mutant in yeast was confirmed by the robust pH-independent signaling of the GPR68–E149Q mutant in the HEK293 model. Together, these two effects result in variant receptors that no longer sense changes in physiological pH below 7.4, as indicated by the gray areas in Figure 3*C*.

The right-shifted pH_50_ values of GPR4–E145Q, GPR65–D286N, and GPR68–E149Q in Figure 3*C* relate directly to mutant pH sensitivity and indirectly to the apparent p*K*_a_ value of the mutated triad residue. This is illustrated by the model in Figure 3*D*, which indicates that at lower and intermediate pH, where all 3 receptors are active, triad p*K*_a_ values are upshifted to be neutral (protonated). Increasing pH further leads to a fully charged triad that inactivates all 3 receptors. Based on our findings in Figure 3*C* and Figure S4*B*, we conclude that the titration of the triad differs slightly in GPR4, GPR65, and GPR68. For GPR65 and GPR68, whose WT receptors have lower pH_50_ values than GPR4, the mutation of Asp and Glu to their neutral Asn and Gln form results in larger ΔpH_50_ upshifts. This outcome indicates that the p*K*_a_ values of the DyaD residue D286 and apEx residue E149 are elevated from 4 to ∼7.0. As we have shown (33, 34), such dramatic p*K*_a_ shifts are the norm for buried acidic residues. In the case of GPR4, the smaller ΔpH_50_ value for GPR4–E145Q indicates that the p*K*_a_ value of apEx residue E145 is right-shifted beyond 7.0. As illustrated in Figure 3*E*, mimicking these p*K*_a_ shifts with Asn and Gln mutations increased the pH_50_ values of all 3 GPCR pH sensors above 7.4 and outside the relevant physiological range of receptor pH sensing. Taken together, these findings validated our computational predictions, confirmed that the buried acidic triad directly regulates GPR4, GPR65, and GPR68 pH sensing, and achieved our objective of nullifying pH sensing while retaining or enhancing receptor function.

### Na^+^ allosterically modulates GPR4, GPR65, and GPR68 pH sensing

Na^+^ are known to be a highly conserved NAM of agonist binding in class A GPCRs (41). Structural studies of have shown that Na^+^ binds to a conserved Asp (D^2.50^) that is also present in the GPR4 (D63), GPR65 (D60), and GPR68 (D67) DyaD site, where the superscript indicates the generic GPCR residue numbering scheme (45). Furthermore, structures solved for proteinase-activated receptors (PAR1 and PAR2) and cysteinyl leukotriene receptor (CLTR1) show Na^+^ chelated by a DyaD site (D^2.50^ and D^7.49^) that is equivalent to the conserved DyaD site in GPR4, GPR65, and GPR68 (refer to Fig. S1*C*) (46, 47, 48). Based on this structural information, we predicted that Na^+^ binding should allosterically modulate GPCR pH sensing by directly regulating the protonation state(s) of DyaD residues.

**Figure 4:**
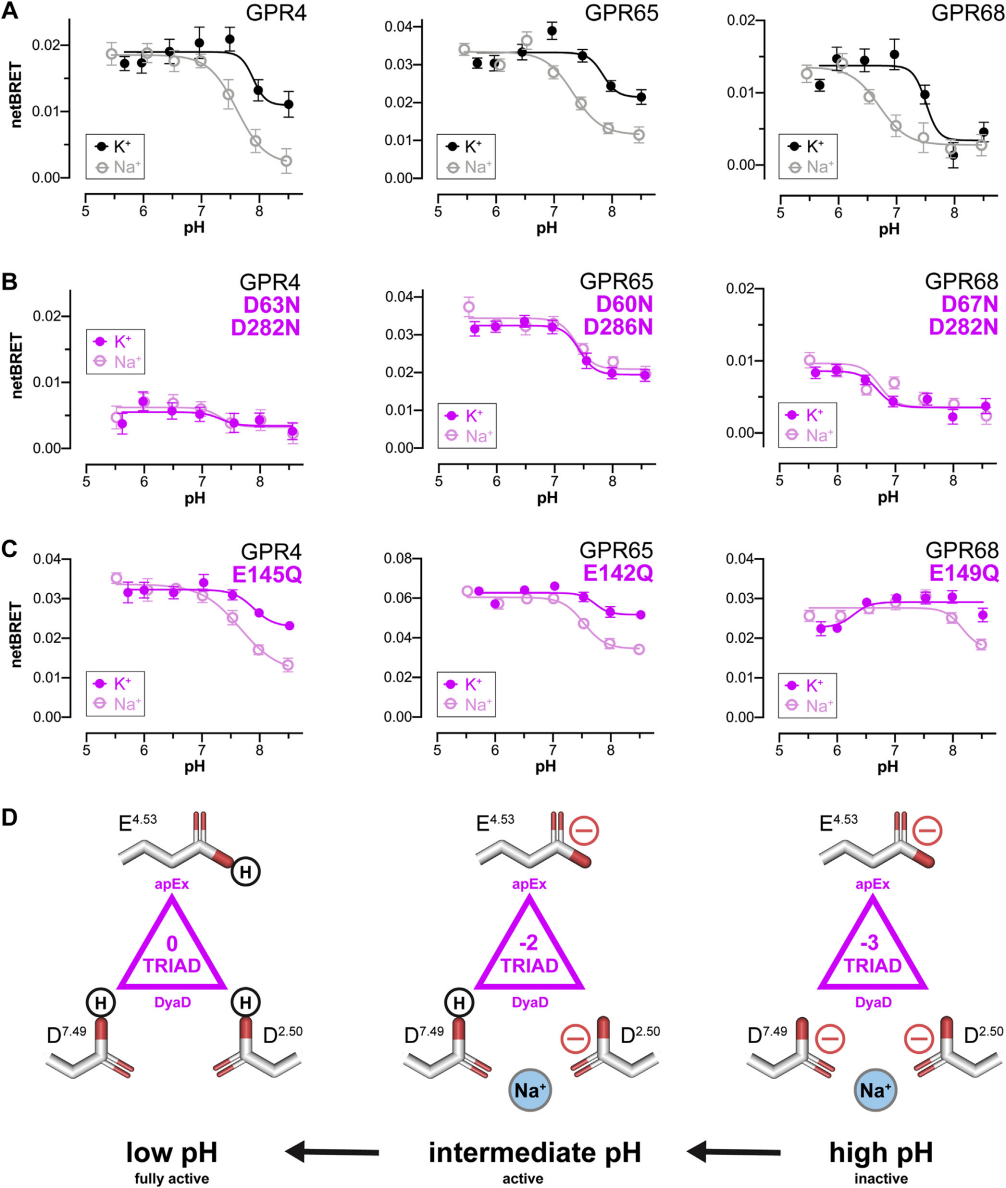
**Na**^**+**^**is an allosteric modulator of GPR4, GPR65, and GPR68 proton sensing.***A*–*C*, pH profiles in the presence and absence of 150-mM K^+^ or Na^+^ for the 3 WT pH sensors (*A*), their DyaD site Asp to Asn double mutants (*B*), and Glu to Gln apEx site single mutants (*C*). Data were collected using mini G protein V-mGsi and are the mean ± SEM of the net BRET signal (*n* = 6–12). For these experiments, slight increases in pH_50_ values were observed as compared with those in Figure 3*C*, which we attributed to the different buffers required for each experiment (see Experimental procedures for details). *D*, interpretive model of triad (de)protonation, GPCR activation, H^+^ and Na^+^ coincidence detection, and Na^+^ negative allosteric modulation as a function of extracellular pH. The net charge and sequence of titration steps and Na^+^ binding are only intended to illustrate the combination of (de)protonation and Na^+^ binding events that underlie the pH-sensing mechanism. BRET, bioluminescence resonance energy transfer; GPCR, G protein–coupled receptor.

Indeed, pH titrations of the 3 receptors in the presence and absence of Na^+^ confirmed this hypothesis (Fig. 4*A*). In the absence of Na^+^, the pH_50_ value of each receptor was dramatically right-shifted with no loss in efficacy at a low pH. As with other GPCRs (41), this result indicates that Na^+^ functions as an NAM by stabilizing the inactive state through interactions with negatively charged (deprotonated) Asp(s) in the DyaD site. In the context of GPR4, GPR65, and GPR68, our experiments indicate that these favorable Coulombic interactions depress/normalize the p*K*_a_ values of D^2.50^ and D^7.49^ to position their pH_50_ values within the most relevant physiological pH range. In contrast, in the absence of Na^+^, the relative dehydration and close proximity of the DyaD site residues appear to reinforce unfavorable self-energies and repulsive Coulombic interactions to cause dramatic p*K*_a_ upshifts favoring the neutral (protonated) states of D^2.50^ and D^7.49^ (33, 34, 35). The direction and magnitude of such p*K*_a_ shifts should give rise to pH profiles that are right-shifted to have pH_50_ values outside the relevant physiologic range, as we observe in Figure 4*A*.

To provide further mechanistic evidence for Na^+^ regulation of DyaD pH sensing, we performed additional experiments using Asp to Asn double mutants of each DyaD site in GPR4, GPR65, and GPR68. As shown in Figure 4*B*, although 2 of the 3 receptors exhibited diminished signaling, each double mutant had pH profiles that were no longer Na^+^ responsive. In contrast, each of the GPR4, GPR65, and GPR68 apEx site (E^4.53^) variants retained Na^+^ sensitivity (Fig. 4*C*), proving that Na^+^ effects on GPCR pH sensing are specific to and mediated directly by the DyaD site. As expected, all 3 double mutants retained varied levels of pH-dependent signaling that appears to unmask the pH-sensing contributions of the apEx site in each receptor. As shown in Figure 4*D*, our collective findings provide a complete understanding of the mechanism of cooperative GPR4, GPR65, and GPR68 pH sensing. At higher pH values, all 3 receptors are maintained in the inactive state by a negatively charged (deprotonated) triad ensemble that is stabilized by Na^+^ binding to the DyaD site. As pH is lowered, the progressive protonation and neutralization of the triad ensemble stabilizes the receptors in the active state. It is the opposing effects of the upshifted triad p*K*_a_ values and the Na^+^ NAM interactions that enable these receptors to detect acidic cues between pH 5 and 7.4. As such, it appears these GPCRs regulate biological activities by functioning as coincidence detectors of H^+^ and Na^+^ signals.

### Cooperative pH sensing emerged in GPR65 and was inherited by GPR4 and GPR68

**Figure 5:**
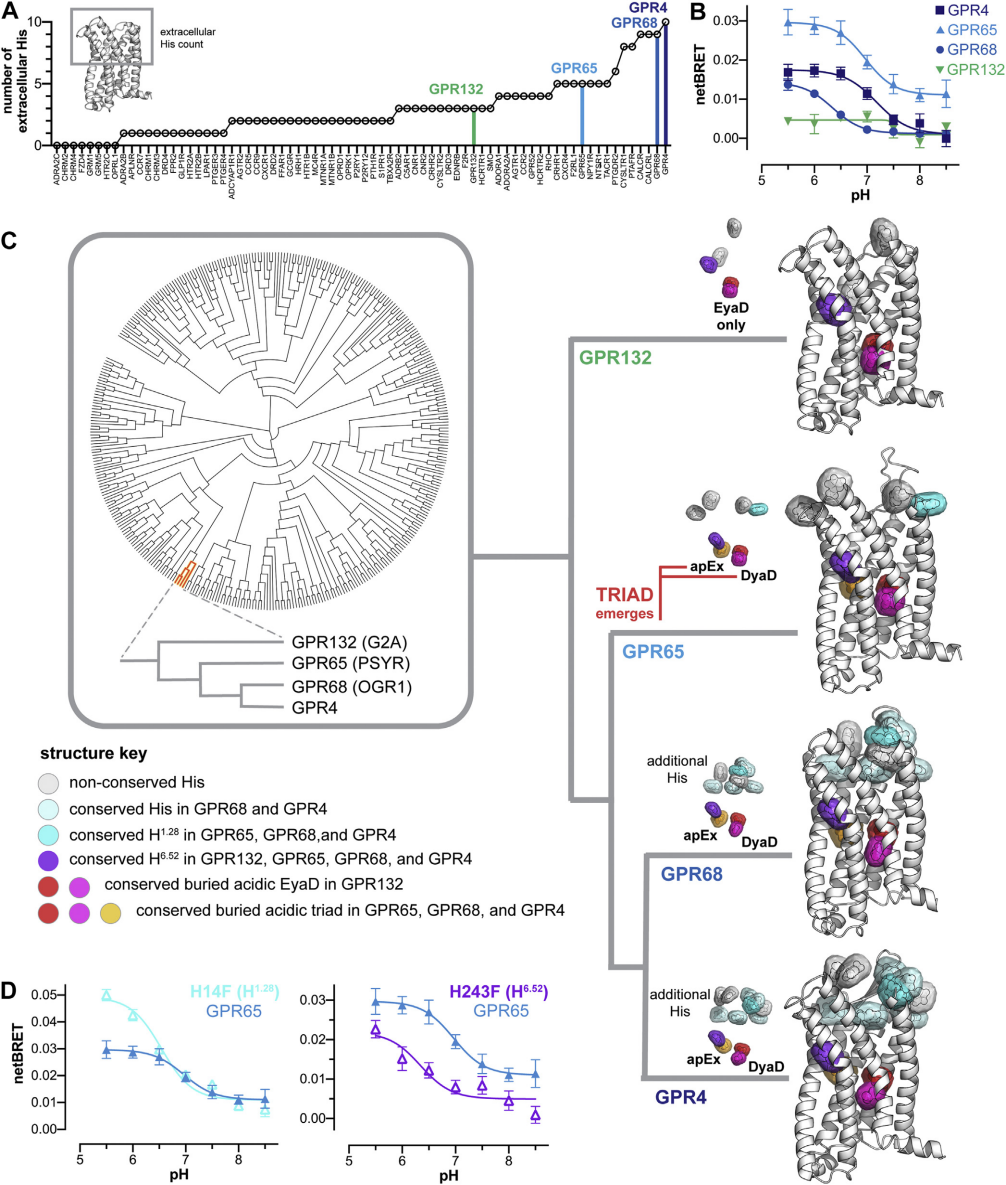
**The molecular evolution of GPR4, GPR65, and GPR68 proton sensing.***A*, ranked number of extracellular His residues in 416 human GPCR structures, represented by 70 nonredundant GPCR genes, and the 4 GPR4, GPR65, GPR68, and GPR132 homology models. *B*, pH profiles of GPR4, GPR65, GPR68, and GPR132 in HEK293 cells measured using the mGp assay. *C*, phylogenetic analysis of 373 nonolfactory GPCRs illustrating the emergence of the buried acidic triad in GPR65 and additional eHis residues in GPR4 and GPR68. The DyaD in GPR132 is an EyaD because the conserved D^2.50^ is E^2.50^. *D*, pH profiles of WT GPR65 (*solid triangles*) and mutations of the conserved eHis residues GPR65–H14F (H^1.28^) and GPR65–H243F (H^6.52^) (*open triangles*). Data shown in panels *B* and *D* were collected using mini G protein V-mGsi and are the mean ± SEM (*n* = 4–9). GPCR, G protein–coupled receptor. eHis, extracellular His; mGp, mini G protein; GPCR, G protein–coupled receptor.

Before this work, it was thought that eHis residues were solely responsible for GPR4, GPR65, and GPR68 proton sensing. However, our findings show that pH sensing is provided primarily by the acidic triads in all 3 receptors. In support of this conclusion, we established two additional sources of informatic and evolutionary insight that further corroborated our mechanistic interpretation. As shown in Figure 5*A*, the number of eHis residues is not predictive of GPR4, GPR65, and GPR68 pH sensing. Although GPR4 (10 eHis) and GPR68 (9 eHis) have many eHis residues, GPR65 contains only 5 eHis residues, a similar number to many other receptors that have not been implicated in proton sensing. These observations led us to investigate the evolutionary origins of GPCR proton sensing by assessing the phylogenetic emergence of eHis and triad residues in the 3 receptors.

As shown in Figure 5, *B*–*C*, phylogenetic analysis shows that GPR4, GPR65, and GPR68 evolved from GPR132 (G2A), which was originally thought to be a cooperative pH sensor (49). However, evidence for GPR132 pH sensing remains limited, inconsistent, and as shown in Figure 5*B*, is not as robust as GPR4, GPR65, and GPR68 pH sensing. In line with this finding, GPR132 lacks a buried acidic triad, which first emerged in GPR65 and was subsequently inherited by GPR4 and GPR68 (Fig. 5*C*). In addition, GPR132 lacks the many eHis residues acquired by GPR4 and GPR68 (Fig. 5*A*). Furthermore, the ancestral pH-sensing GPCR, GPR65, also lacks the additional eHis residues acquired by GPR4 and GPR68, indicating these residues are secondary modulators of triad proton sensing. This conclusion is further supported by His to Phe mutations of GPR65 at the only two sites conserved in all 3 GPCRs, H^1.28^ (GPR65–H14F) and H^6.52^ (GPR65–H243F). As shown in Figure 5*D*, the GPR65–H243F mutation diminished the receptor function, as was the case in GPR4 and GPR68 (Fig. S4*D*), and the GPR65–H14F mutation improved the dynamic range of triad proton sensing. If these conserved His residues were not responsible for the emergence of pH sensing in GPR65, they cannot be responsible for the primary mechanism of proton sensing by GPR4 and GPR68. Indeed, the consequences of H^1.28^ and H^6.52^ mutations in GPR4 and GPR68 were variable and typically diminished or did not affect receptor function (Fig. 3*B* and Fig. S4*D*). If conservation of H^1.28^ and H^6.52^ was essential for pH sensing, we would have expected similar results for all 3 receptors.

Considering further the emergence of pH sensing in GPR65, it might be expected that GPR132, the evolutionary ancestor of GPR65, could be converted into a cooperative pH sensor by installing the missing apEx site. However, mutating V^4.53^ to E^4.53^ in GPR132 failed to produce a pH-sensing triad. Based on this finding and on the evolutionary sequence of events (Fig. 5*C*), we conclude that cooperative pH sensing in humans began with the emergence of the acidic triad in GPR65 and that additional eHis residues acquired by GPR4 and GPR68 either modulate triad pH sensing or fulfill otherwise unknown biological functions. One possibility is that these eHis residues modulate GPCR pH sensing by mediating ionic signals (50). Another possibility is that GPR4 and GPR68 use their multitude of eHis residues to recruit unknown anionic peptide or protein ligands.

### New *in vitro* and *in vivo* opportunities

With a complete understanding of GPR4, GPR65, and GPR68 pH sensing in hand, new avenues of biological inquiry are now open for exploration. From an evolutionary perspective, we have already identified other GPCRs that contain DyaD sites and are beginning to test whether these receptors are one evolutionary step from sensing pH. From a biological and clinical perspective, our work provides new pH-insensitive versions of GPR4, GPR65, and GPR68 that will enable the broader community to quantify the importance of GPCR pH sensing in a variety of cellular and physiological systems, including specific cell, organoid, tumor, and animal models. We envision this would be done by comparing the effects of WT and pH-insensitive receptor variants to assess the biological ramifications of GPR4, GPR65, and GPR68 signaling in the presence and absence of acidic cues. Similarly, from the perspective of drug discovery and deorphanization, given the pH-independent signaling of our variant collection, we can now circumvent the stress of extracellular acidification to develop scalable cell-based screening assays for inhibitors, inverse agonists, and other pharmacological tools. Together, we believe these new advantages will further illuminate the dark pharmacology of GPR4, GPR65, and GPR68 and significantly advance our understanding of GPCR pH sensing in biology, health, and disease.

## Experimental procedures

### GPCR structures and homology models

Sequences of 164,174 structures were downloaded from the Protein Data Bank (PDB, https://www.rcsb.org) and filtered for human GPCRs using BLAST+ (the National Center for Biotechnological Information) and a local library of human 373 GPCR gene sequences. The resulting collection of 416 GPCR structures was aligned using PyMOL (Schrödinger, New York, NY) to the reference GPCR structures of bovine rhodopsin (PDB code: 1F88, chain A). Homology models for GPR4, GPR65, GPR68, and GPR132 were downloaded from the GPCR-HGmod database (51). In addition, GPR4, GPR65, GPR68, and GPR132 homology models were calculated using Swiss-Model (https://swissmodel.expasy.org). Within the transmembrane helix domains, the i-Tasser (51) and Swiss-Model structures were indistinguishable. All pHinder calculations were performed on the i-Tasser homology models.

### pHinder calculations

The pHinder algorithm has been described in detail previously (40). Briefly, a pHinder calculation for identifying buried ionizable residues is performed in two phases. In the first phase, all of the ionizable residues are triangulated. This triangulation is then minimized and trimmed of any edges that exceed 10 Å. The result is a network data structure containing all ionizable residues in a protein. In the second phase, the Cα atoms of the protein are used to calculate a protein surface comprising interconnected triangular facets. This surface is used to calculate the average depth of each ionizable residue *via* the closest surface facet and its neighbors. In a typical pHinder calculation, each residue is classified as core (≥−3.0 Å below the surface), margin (<−3.0 Å below the surface and ≤1.05 Å above the surface), or exposed (>1.05 Å above the surface). Using this classification system, we trim the triangulated network of ionizable residues, leaving behind only those network nodes that are buried in the protein. In cases where a core residue shares a network edge with a margin or exposed ionizable residues, these residues are also included in the network visualization. This process is summarized in Figure S1*A*.

### Analysis of spatially conserved buried ionizable residues

pHinder calculations were performed on the aligned set of 416 human GPCR structures. The core networks from each calculation were superimposed, and network nodes were clustered using a procedure we developed previously called the consensus network analysis (CNA) (37). Briefly, in the CNA algorithm, the full collection of network nodes is triangulated. The triangulation is then trimmed using an edge distance cutoff typically between 0.5 Å and 3.0 Å (in this case 1.5 Å). As shown in Figure S1*B*, this process identifies tight clusters of spatially conserved nodes within the overall triangulation. For these calculations, we elected to do two rounds of clustering. In the first round, we identified the network clusters and found that many contained repeated contributions from redundant GPCRs in the set of 416 structures. In the second round, we removed any redundant contributions and redid the triangulation and clustering using the same distance edge constraint of 1.5 Å and a minimum cluster size of 5. The results are shown in Figure S1*B*.

### Analysis of spatially conserved Na^+^

All Na^+^ associated with the 416 human GPCR structure were collected and clustered by CNA using an edge distance cutoff of 2.0 Å minimum cluster size of 5. 40 of the 416 GPCR structures representing 7 receptor genes contributed Na^+^ to this analysis: ADORA2A, CLTR1, DRD4, PAR1, PAR2, HCRTR1, and OPRD1. Representative results are shown in Figure S1*C* for the crystal structure of human proteinase–activated receptor 1 (PDB code: 3VW7, chain A) (48).

### Counting surface-exposed His residues

Three Cα atoms from the aligned bovine rhodopsin reference (Cα atoms for residues 52, 163, and 214 of PDB code: 1F88, chain A) were used to define a horizontal plane located at the midpoint of the lipid bilayer. His residues located above this plane were classified as extracellular. In the cases where a GPCR was represented by more than one structure, results were reported for the structure with the greatest number of eHis residues.

### Phylogenetic analysis

Phylogenetic analysis of the set of 373 nonolfactory receptors was performed using Clustal Omega (https://www.ebi.ac.uk/Tools/msa/clustalo/) (52) and visualized using Dendroscope (53).

## Reagents

All media, buffers, and solutions are described in Supporting Data Set 1. Those requiring pH adjustments were measured using an Accumet XL150 pH meter (Fisher Scientific, Hampton, NH).

### Cell lines

Yeast DCyFIR strains expressing human GPR4, GPR65, or GPR68 have been described previously (42). The complete list of 430 yeast strains constructed and used throughout this study can be found in Supporting Data Set 2. HEK293T cells (HEK293T/17) were purchased from the American Type Culture Collections (ATCC CRL-11268; Gaithersburg, MD).

### Plasmids and receptor constructs

All CRISPR plasmids constructed throughout this study were derived from pML104 (54). The name, sequence, and other relevant information are provided in Supporting Data Set 3. WT GPR4–Rluc8, GPR65–Rluc8, GPR68–Rluc8, and GPR132–Rluc8, along with the set of nuclear export sequence (NES)–Venus–mG (V-mG) constructs, were a gift from Nevin Lambert. Details for these, along with the various mutant GPCR–Rluc8 constructs generated throughout this study, are also provided in Supporting Data Set 3. All plasmids were maintained in *Escherichia coli* strain DH5α (New England BioLabs, Ipswich, MA) and purified using the EZ Plasmid Miniprep Kit (EZ BioResearch, St Louis, MO) or ZymoPURE II Plasmid Midiprep Kit (Zymo Research, Irvine, CA).

### DCyFIR strain engineering for DVP of GPR4, GPR65, and GPR68

#### CRISPR plasmid library design

Using pML104, 34 new CRISPR constructs were generated, each containing different guide RNA sequences for targeting desired mutation sites within GPR4, GPR65, and GPR68. Briefly, this was performed using a modified site-directed mutagenesis approach, where inverse PCR was performed using a universal reverse oligo (5’-phosphorylated) accompanied by a forward oligo containing a 20-bp 5’ overhang with the guide RNA. Once the linearized plasmid PCR product was ligated, transformed, and purified, the newly introduced guide sequence was confirmed *via* Sanger sequencing (Eurofins Genomics, Louisville, KY).

#### Mutagenesis approach and design of DNA payload

The CRISPR/Cas-9 system was used to introduce point mutations at specified sites within the desired human GPCR genes installed in our yeast DCyFIR strains. Mutations were introduced by transforming yeast with a specified CRISPR plasmid (described above) along with a 60- to 100-bp oligonucleotide insert homologous to the cut site but containing (1) a new codon encoding the desired amino acid and (2) a single-base substitution removing the protospacer adjacent motif site. These oligonucleotide inserts were designed to have 30- to 50-bp homology arms flanking the codon of interest.

#### Yeast transformation procedure

For initially introducing mutations, the base GPCR-G_αi_ and -G_αz_ DCyFIR strains of the specified WT receptors were prepared and transformed as previously described (43), with the following exceptions: 1-nmol DNA template (*i.e.*, oligonucleotide insert described above) (55) and 150-ng CRISPR plasmid were used. Genomic DNA from select transformants was extracted and used for identifying successful mutants *via* Sanger sequencing. Sequence-confirmed mutant genes were then transformed into all ten base G_α_ DCyFIR strains, integrations confirmed *via* PCR, and strains stored as glycerol stocks (43).

### Using DVP to identify pH-sensing residues in GPCRs

#### Profiling GPR4, GPR65, and GPR68 mutants at pH 5.0

Strains were struck onto yeast extract peptone dextrose plates and grown for 1 to 2 days at 30 °C. Two colonies per strain were picked into 96-well deep-well blocks (Greiner Bio-One; catalog no. 780271-FD) containing 500 μl of the synthetic complete dextrose (SCD) screening medium, pH 6.5. Cultures were grown at 30 °C until log-phase growth was reached (*A*_600_ _nm_ = 0.2–1.5; typically, 12–13 h), at which point 200-μl aliquots were transferred to a 96-well plate(s) (USA Scientific; catalog no. CC7672–7596), centrifuged (3000*g* for 5 min), harvested, and resuspended in 200-μl the SCD screening medium, pH 5.0. A Biomek NX^P^ liquid-handling robot was used to prepare 50 μl of cell cultures normalized to an *A*_600 nm_ of 0.05 in black 384-well clear-bottom plates (Greiner Bio-One; catalog no. 781096). The plates were covered with a porous film (Diversified Biotech; catalog no. BERM-2000), shaken (1200 rpm for 30 s) on a MixMate microplate shaker (Eppendorf, Hamburg, Germany), and incubated at 30 °C for ≈22 h. Fluorescence (instrument gain of 1300) and *A*_600 nm_ measurements were taken at 2 to 3 h increments.

#### Profiling active mutants at pH 7.0

Based on profiling at pH 5.0, active strains (*i.e.*, those exhibiting higher fluorescence than that of the respective G_α_ DCyFIR strain containing no receptor) were prepared as described above. Once cultures in the SCD screening medium, pH 6.5, had reached log-phase growth, 200-μl aliquots were transferred to 96-well plates, centrifuged (3000*g* for 5 min), harvested, and resuspended in the SCD screening medium, pH 7.0. These cultures were used to prepare new 200-μl cultures normalized to an *A*_600 nm_ of 0.05 in a 96-well format using a Biomek NX^P^ liquid-handling robot. Plates were covered with porous film, shaken (1200 rpm for 30 s), and incubated at 30 °C for 20 h. After this time, fluorescence (instrument gain of 1200) and *A*_600 nm_ measurements were taken over a linear dilution series.

### pH titrations of WT GPR4, GPR65, and GPR68 in yeast

WT GPR4–G_αi_, GPR65–G_αi_, and GPR68–G_αi_ DCyFIR strains were struck onto yeast extract peptone dextrose plates and grown for 1 to 2 days at 30 °C. Four colonies per strain were picked into 96-well deep-well blocks containing 1.2 ml of the SCD screening medium at pH 6.0. Cultures were grown at 30 °C to the log phase (*A*_600 nm_ = 0.5–1.5; 14–16 h) and subsequently used to prepare 1-ml growths normalized to an *A*_600 nm_ of 0.5 in a 96-well deep-well format. These normalized cultures were used to deliver ten 80-μl aliquots into a 96-well plate, which was then centrifuged (3000*g* for 5 min) and harvested, and cells were resuspended in 80-μl pH-specific SCD screening medium (*i.e.*, pH 5.0, 5.5, 5.75, 6.0, 6.25, 6.5, 6.75, 7.0, 7.25, or 7.5). The plate was shaken (1200 rpm for 30 s) and 50-μl aliquots were transferred to a black 384-well clear-bottom plate, covered with porous film, placed at 30 °C, and incubated for 20 h. After an initial equilibration period of 5 to 7 h, fluorescence (instrument gain of 1200) and *A*_600 nm_ measurements were taken at 2 to 3 h increments.

### Data acquisition and analysis in yeast

DVP data and pH titrations were collected from 2 and 4 biological replicates, respectively. Absorbance (22 flashes/well; excitation filter, 600 nm) and mTq2 fluorescence (bottom read; 10 flashes/well; excitation: 430/10 nm, dichroic filter: LP 458 nm, emission filter: 482/16 nm) were measured using a CLARIOstar multimode microplate reader (BMG LabTech, Offenburg, Germany). Data were collected over time (pH 5.0 screens and pH titrations) or *via* linear dilution (pH 7.0 screens) and fit in GraphPad Prism 8.3.0 (San Diego, CA) to generate slope and intercept values, which were used to extrapolate fluorescence to a standardized *A*_600 nm_ value of 1.0. Where applicable, error bars represent the standard error (Prism’s equivalent to SD) of the fitted slopes.

### Mutant receptor constructs for BRET-based GPCR assays in HEK293 cells

Using WT GPR4–Rluc8, GPR65–Rluc8, GPR68–Rluc8, and GPR132–Rluc8 plasmids, our mutant library for mammalian expression was constructed using a modified site-directed mutagenesis approach, as described in the CRISPR plasmid library design section. For this purpose, each mutant construct required (1) a reverse oligo (5’-phosphorylated) designed to anneal directly upstream of the mutagenesis site and (2) a forward oligo designed to anneal directly downstream of the mutagenesis site but containing a 3-bp 5’ overhang with the codon encoding the desired amino acid substitution. This PCR scheme resulted in the simultaneous deletion/introduction of the WT/mutant codon. All mutant GPCR–Rluc8 constructs were validated *via* Sanger sequencing.

### Cell culture and transient transfection

HEK293 cells were maintained in the Dulbecco's modified Eagle's medium supplemented with 10% fetal bovine serum and 1% penicillin–streptomycin. Before transfections, cells were seeded into 6-well plates (USA Scientific; catalog no. CC7682–7506) at a density of 3 × 10^5^ cells/well. Once cells reached confluency, the growth medium was replaced and transfections were performed using linear polyethylenimine hydrochloride (MW 40k; Polysciences, Inc, Warrington, PA) to cotransfect the GPCR–Rluc8 plasmid (0.5 μg) and one of four mGps: NES–Venus–mGs, NES–Venus–mGsi, NES–Venus–mGsq, or NES–Venus–mG12 (2.0 μg) (44). Total transfected DNA was adjusted to 3.0 μg in each well using pcDNA3.1(+). Cells were transfected for 2 to 2.5 h and allowed to recover for 22 to 24 h before experimentation.

### BRET mGp assay

#### BRET and luminescence measurements for characterizing pH behavior

Cells expressing the GPCR–Rluc8 and NES–Venus–mG constructs were washed twice with 2-ml Dulbecco's phosphate-buffered saline, resuspended *via* trituration with 1-ml Hank's balanced salt solution, and centrifuged (600*g* for 3 min) and resuspended in a final 150-μl Hank's balanced salt solution. Using resuspended cells, ∼1.6 × 10^5^ cells/well (*i.e.*, 20 μl) were added to opaque black 96-well plate(s) (Greiner Bio-One; catalog no. 655209) containing 130-μl pH-specified BRET assay buffer. Once all cells had been added to a given plate, 50 μl of 30-μM h-coelenterazine (Nanolight Technologies; 7.5 μM final) was added. 30-μM h-coelenterazine solutions were prepared using pH-specific assay buffers. Data were collected immediately after addition of h-coelenterazine. Unless specified, all BRET-based measurements were performed with NES–Venus–mGsi to be consistent with our yeast-based pH titrations.

#### BRET-based measurements for evaluating the role of Na^+^

Cells expressing the GPCR–Rluc8 and NES–Venus–mGsi constructs were washed twice with 2-ml K^+^-PBS, pH 7.0, and resuspended in 1 ml *via* trituration. Cells were then centrifuged (600*g* for 3 min) and resuspended in a final 150-μl, and ∼8.0 × 10^4^ cells (*i.e.*, 10 μl) were added per well to opaque black 96-well plate(s) containing 140-μl pH-specified K^+^- or Na^+^-PBS. Once all cells had been added to a given plate, 50 μl of 30-μM h-coelenterazine (7.5-μM final) was added, which was prepared using pH-specific K^+^-PBS or Na^+^-PBS. Data were collected immediately after h-coelenterazine addition. Before assays, the pH of all K^+^-PBS and Na^+^-PBS buffers was measured and recorded.

#### BRET measurements and data analysis

A CLARIOstar multimode microplate reader (top read; instrument gain of 3500; emission filters: 482/16 nm and 520/10 nm) was used for luminescence and BRET measurements. Raw BRET was calculated as the emission intensity of the BRET acceptor (*i.e.*, NES–Venus–mG; 520 nm) divided by that of the BRET donor (*i.e.*, GPCR–Rluc8; 482 nm). Net BRET values were determined by subtracting this ratio from the raw BRET of cells only expressing the BRET donor. GraphPad Prism 8.3.0 was used for data analysis. For pH titrations, data were fit using the four-parameter pharmacological function log(agonist) *versus* response. Reported pH_50_ values are equivalent to the negative logarithm of the half-maximum effective concentration and have been provided in Supporting Data Set 4.

## Data availability

All relevant data, protocols, and results of analyses are included in the main text and within the Supporting Information and data sets. Computer code, yeast strains, and mammalian plasmids are available upon request.

## Conflict of interest

The authors declare that they have no conflicts of interest with the contents of this article.

## Supporting information

Figures S1–S4

Supporting Data Set 1

Supporting Data Set 2

Supporting Data Set 3

Supporting Data Set 4
