# Supplementary material for: The evolution and mechanism of GPCR proton sensing
Source: J Biol Chem. 2020 Dec 13;296:100167. doi: 10.1074/jbc.RA120.016352 (PMC7948426; doi:10.1074/jbc.RA120.016352)
Supplement: Supporting Data Set 3 [file mmc4.pdf]

Supporting Information for

The evolution and mechanism of GPCR proton sensing

Jacob B. Rowe[1], Nicholas J. Kapolka[1], Geoffrey J. Taghon[1], William M. Morgan[1], and Daniel G. Isom[1,2,3,*]

From the [1]Department of Molecular and Cellular Pharmacology, University of Miami Miller School of Medicine, Miami, FL, 33136; [2]University of Miami Sylvester Comprehensive Cancer Center, Miami, FL, 33136; and [3]Institute for Data Science Computing, University of Miami, Coral Gables, FL, 33146

*Corresponding Author: Daniel G. Isom, PhD

E-mail: disom@miami.edu

This PDF file includes:

Figures S1 to S4
Legends for Datasets S1 to S4

Other Supporting Information for this manuscript include the following:

Datasets S1 to S4

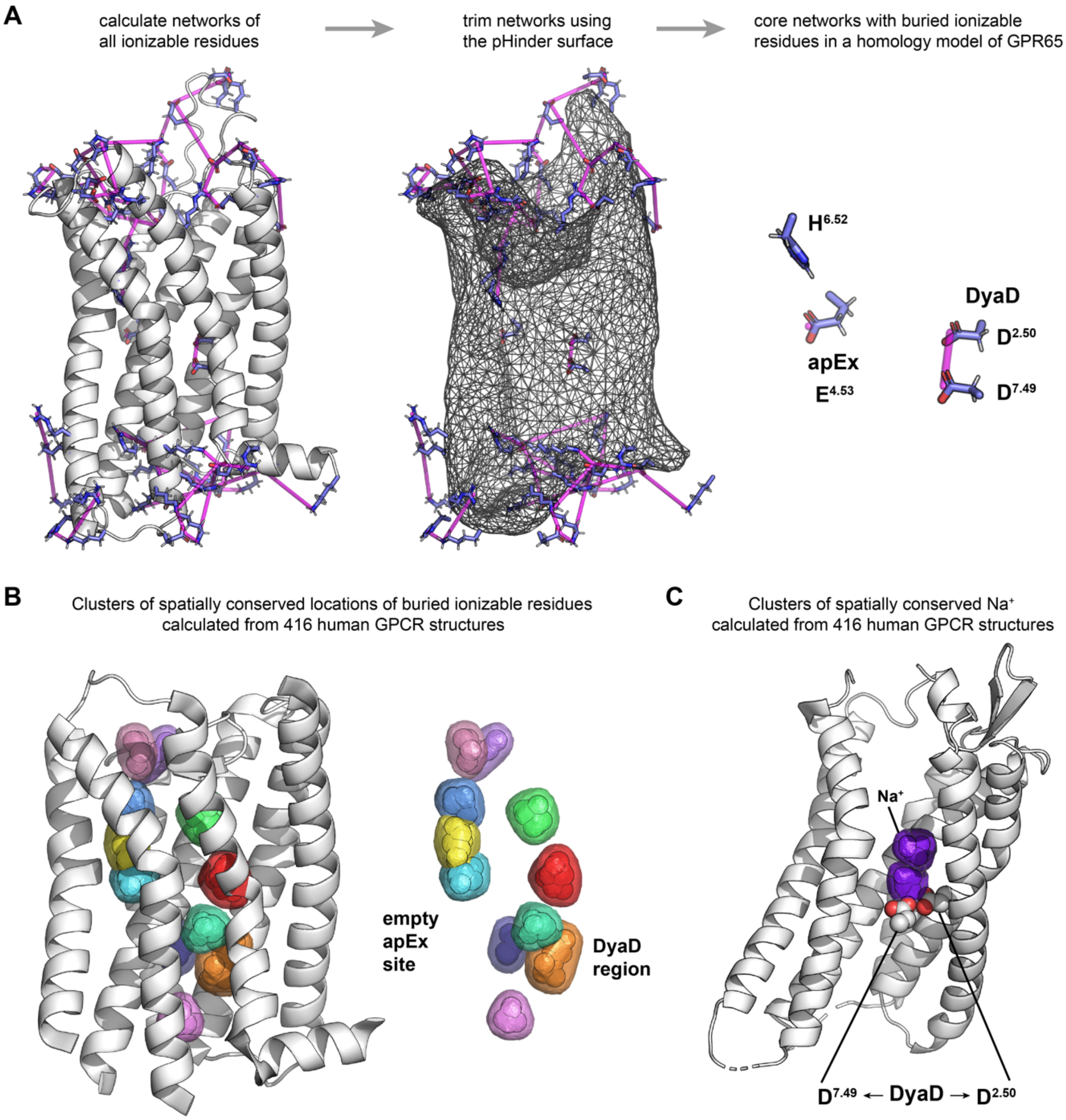

Figure S1. Structural informatics of GPCR pH sensing (related to Fig. 1). *A,* The major steps of the pHinder algorithm for identifying buried ionizable networks in a representative homology model of GPR65. *B,* Spatial conservation of the location of buried ionizable residues in 416 human GPCR structures indicating the DyaD region and the empty apEx site that comprise the buried acidic triad in GPR4, GPR65, and GPR68. Colors represent the individual clusters identified by the CNA algorithm. *C,* Spatially conserved Na^+ ion clusters associated with the 416 human GPCR structures illustrated in the human proteinase-activated receptor 1 (PDB code 3VW7, chain A). 40 of the 416 GPCR structures, representing 7 non-redundant receptor genes, contributed Na^+ ions to our CNA analysis: ADORA2A, CYSLTR1, DRD4, F2R, F2RL1, HCRTR1, and OPRD1. Transmembrane helix 7 has been cut away to reveal the DyaD and Na^+.

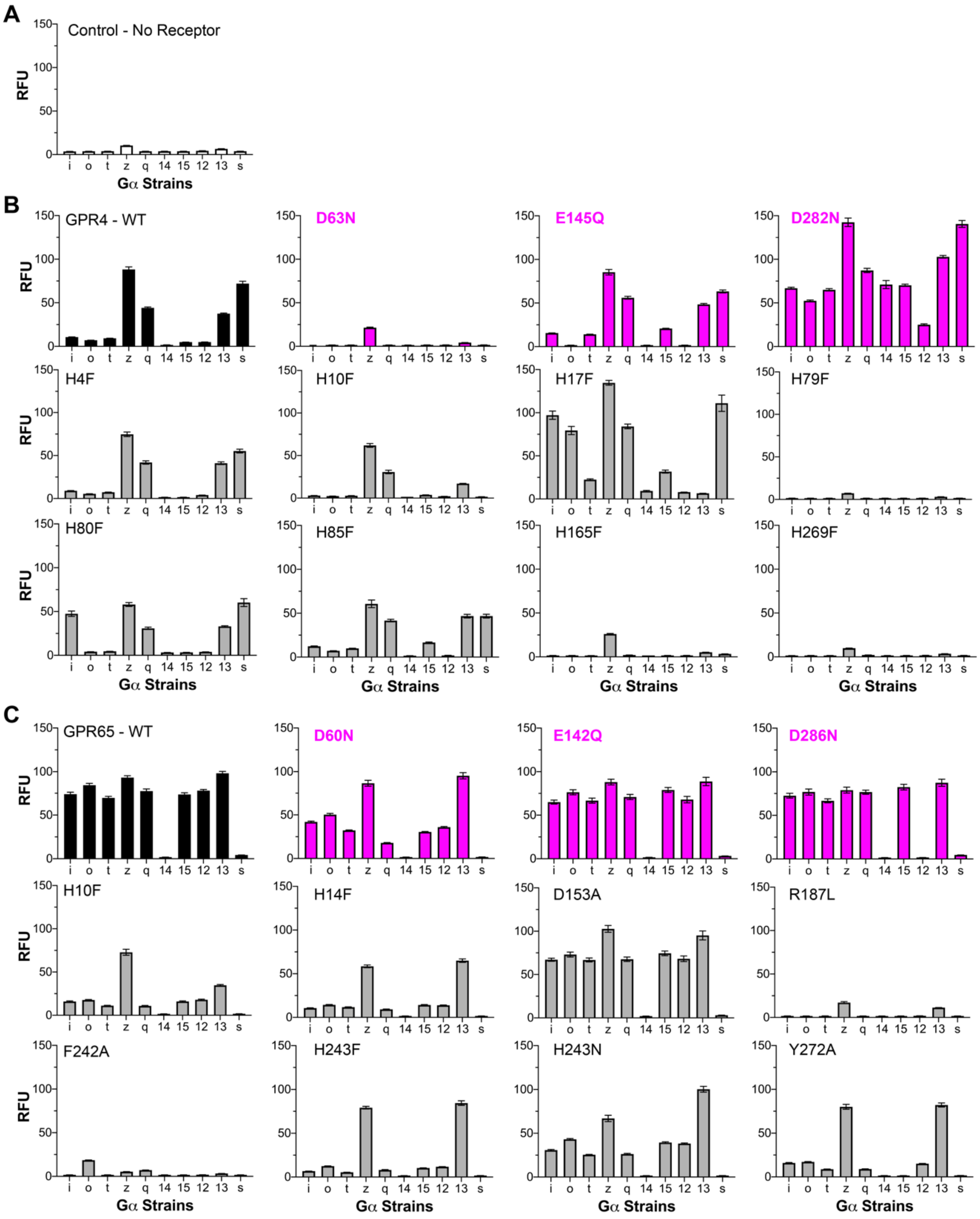

A
Control - No Receptor
RFU
Gα Strains
B
GPR4 - WT
D63N
E145Q
D282N
H4F
H10F
H17F
H79F
H80F
H85F
H165F
H269F
C
GPR65 - WT
D60N
E142Q
D286N
H10F
H14F
D153A
R187L
F242A
H243F
H243N
Y272A
i o t z q 14 15 12 13 s

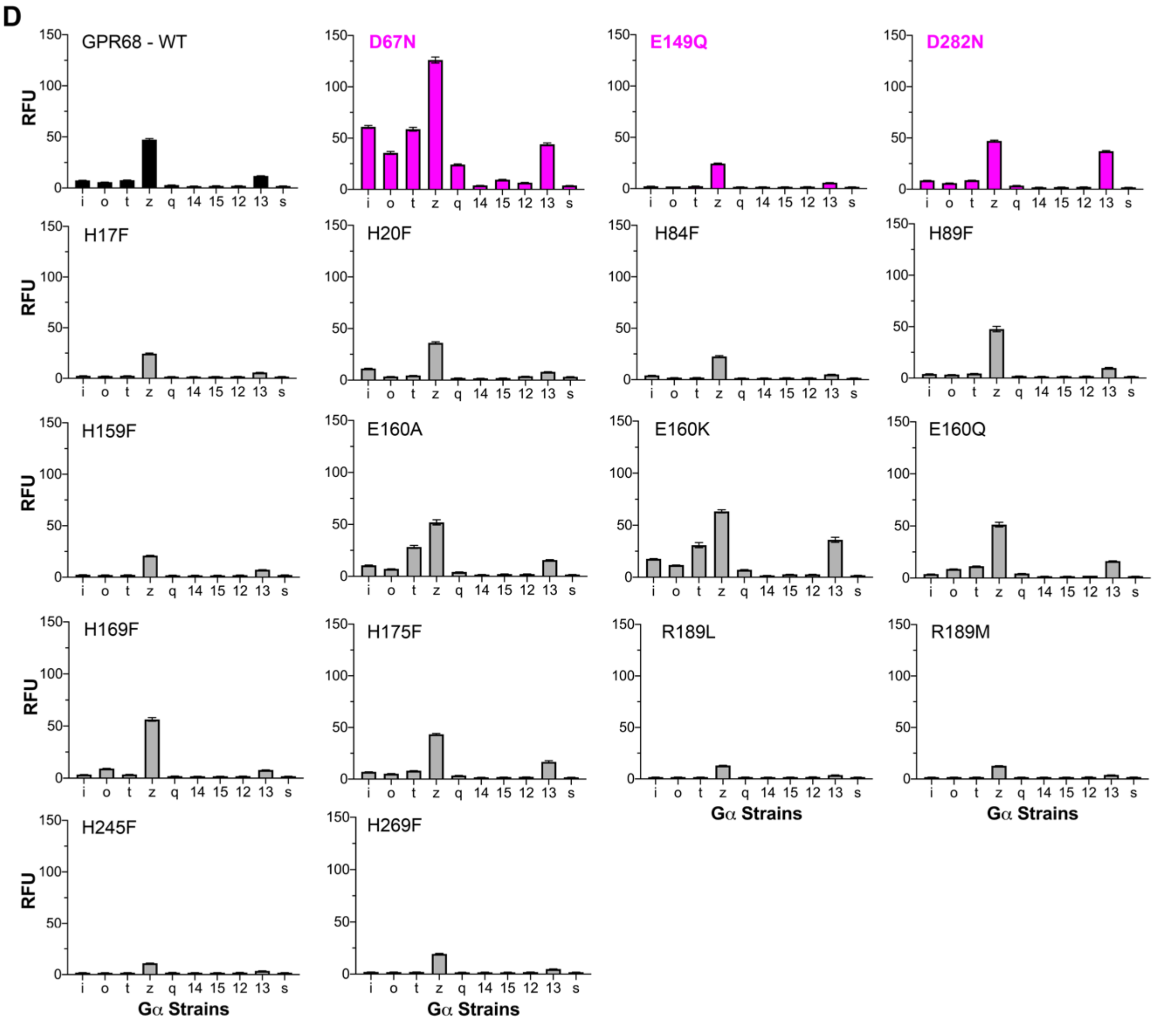

Figure S2. DVP of 420 DCyFIR strains to assess viability at pH 5.0 (related to Fig. 2). *A-D*, DVP results at pH 5.0 for 10 control strains with no receptor (*A*), 120 GPR4-related strains (*B*, 10 wild-type and 110 mutants), 120 GPR65-related strains (*C*, 10 wild-type and 110 mutants), and 180 GPR68-related strains (*D*, 10 wild-type and 170 mutants). Data are mean relative fluorescence (RFU) of strains corresponding to no receptor controls (*white*), wild-type receptors (*black*), triad mutant receptors (*pink*), and rSites mutant receptors (*gray*). Error bars represent s.d. (n = 2).

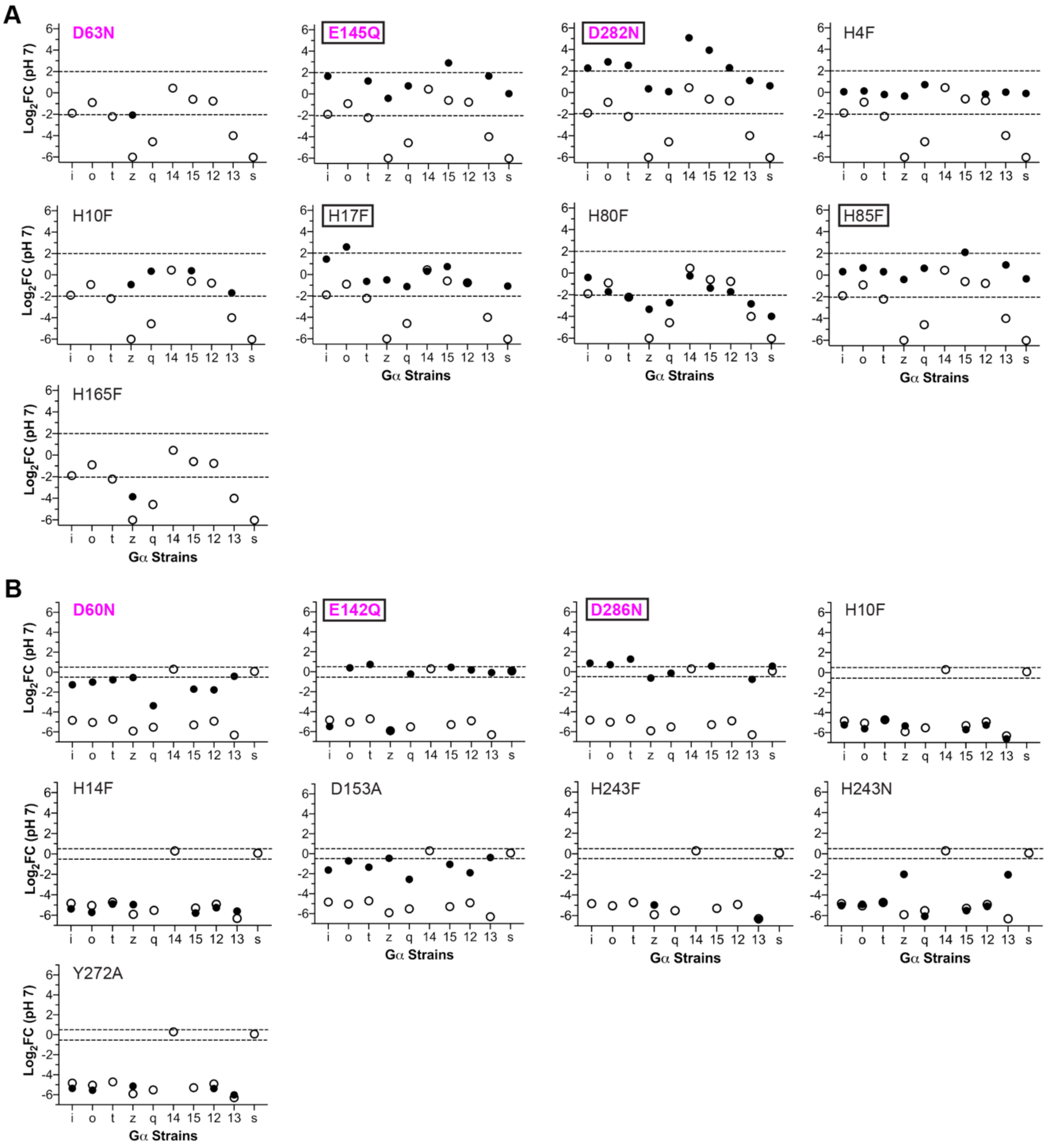
A
D63N
E145Q
D282N
H4F
H10F
H17F
H80F
H85F
H165F
B
D60N
E142Q
D286N
H10F
H14F
D153A
H243F
H243N
Y272A
Log2FC (pH 7)
Gα Strains
i o t z q 14 15 12 13 s

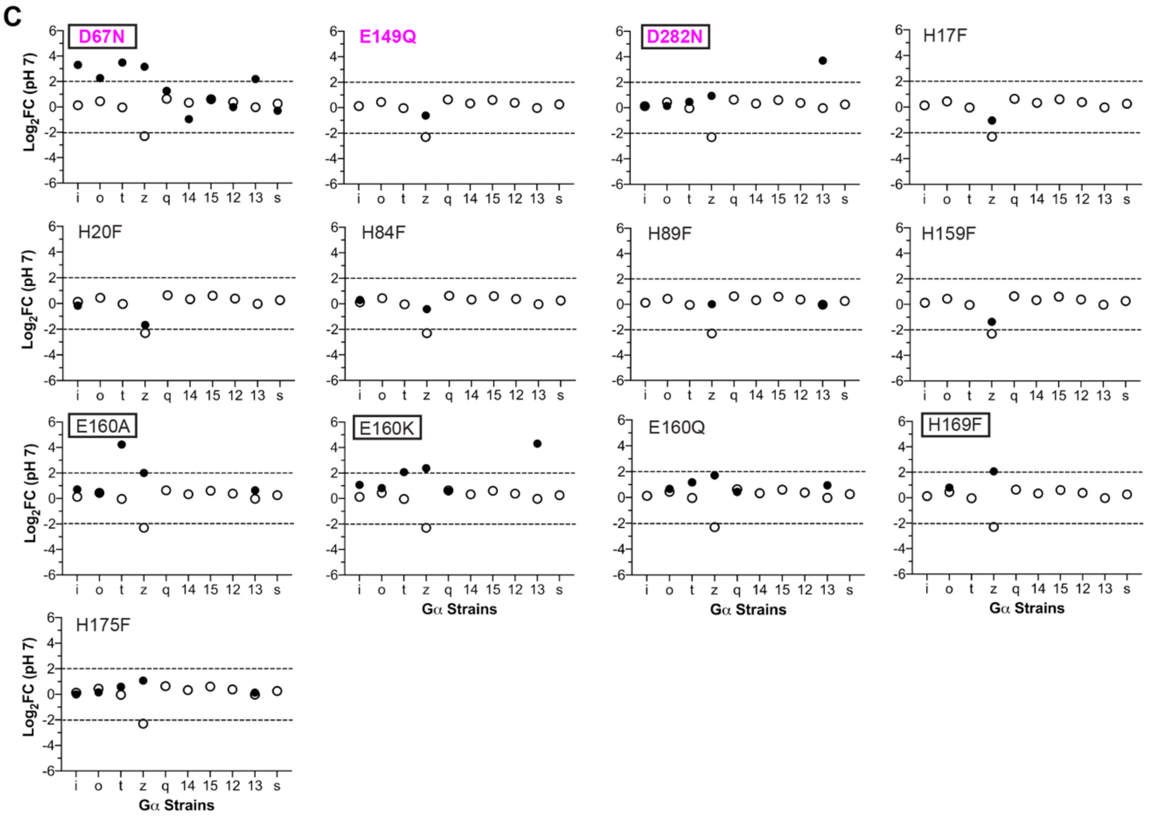

Figure S3. DVP of the 195 viable GPR4, GPR65, and GPR68 mutants at pH 7.0 (related to Fig. 2). *A-C*, Functional triad (*pink*) and rSite mutant DCyFIR strains (Figs. 2*B-C* and S2) of GPR4 (*A*), GPR65 (*B*), and GPR68 (*C*) profiled at pH 7.0. Results were scored as $\log_2$ fold change (FC) in mTq2 fluorescence relative to each corresponding wild-type DCyFIR strain (*black circles*; $n = 2$). Open circles represent control DCyFIR strains with no receptor ($n = 2$). Thresholds of $\log_2$ FC > 2.0 (GPR4 and GPR68) or > 0.5 (GPR65) (*top dashed line*) were set for identifying mutants with one or more DCyFIR strains exhibiting increased signaling (*black boxes*). The remaining DCyFIR-strain mutants were characterized as having no change in signaling ($\log_2$ FC $\pm$ 2.0 for GPR4 and GPR68; $\log_2$ FC $\pm$ 0.5 for GPR65) or decreased signaling ($\log_2$ FC < -2.0 for GPR4 and GPR68; $\log_2$ FC < -0.5 for GPR65). Strains with the same $\log_2$ FC values as those of the control DCyFIR strains were characterized as having no detectable signaling or Gα coupling under the specified conditions.

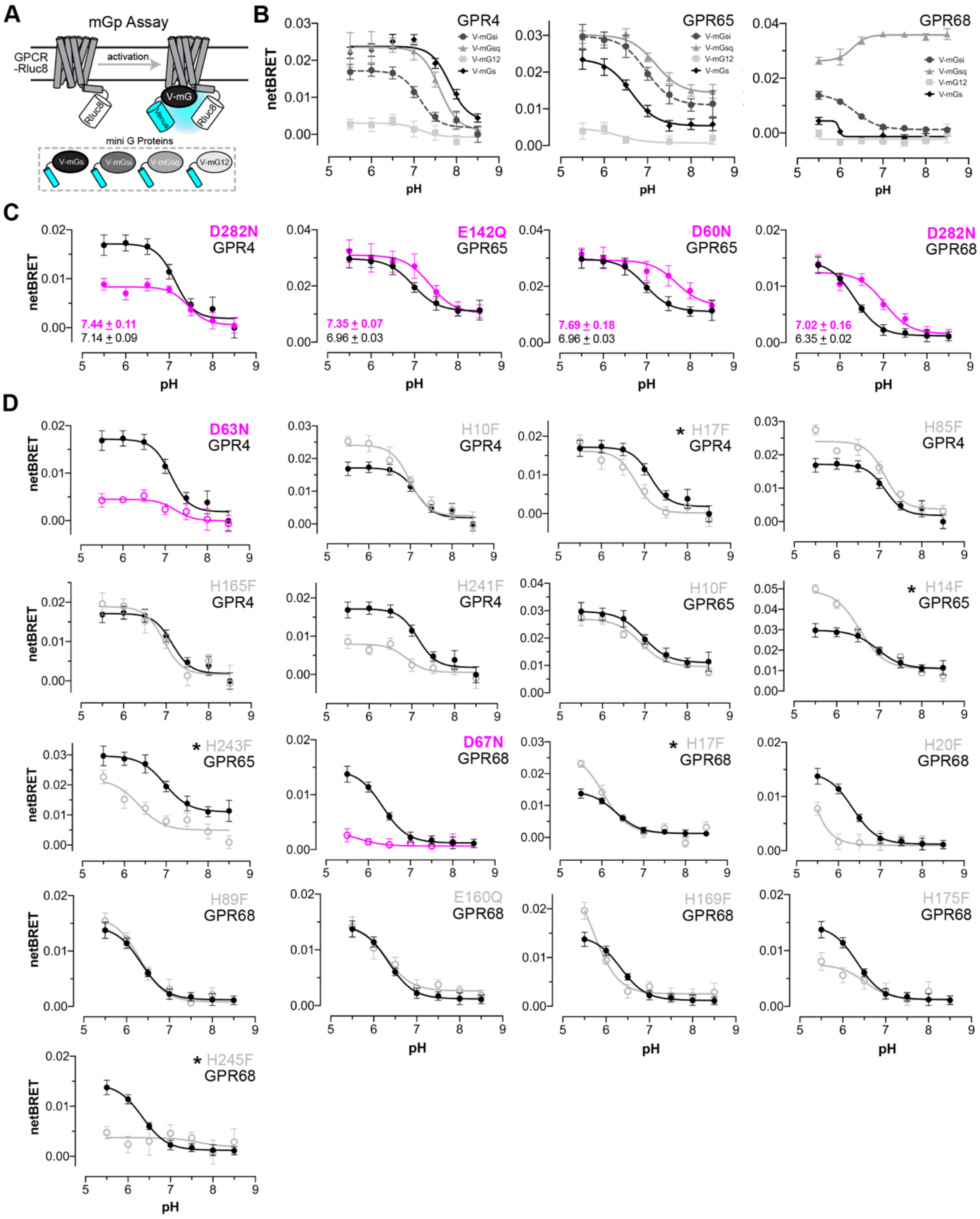

Figure S4. Additional pH profiles of GPR4, GPR65, and GPR68 mutants measured using the mGp assay (related to Figs. 3 and 5). *A,* Schematic illustrating the BRET-based mini G protein (mGp) assay used in HEK293 cells. Coupling of a Venus-tagged mGp (V-mG) to a luciferase-tagged GPCR (GPCR-Rluc8) results in BRET. Four different mGp were used for this study to cover the four major families of Gα coupling combinations (V-mGs, V-mGsi, V-mGsq, V-mG12). *B,* pH profiles of wild-type GPCR pH sensors using all four mini G proteins. Dashed lines indicate the mGp used for mutational analyses (V-mGsi). *C,* Additional pH profiles of triad mutants (*pink*) that exhibited upshifted pH_{50} values. Inset values are the pH_{50} values (mean ± s.e.m.) for each triad

variant and the corresponding wild-type receptor. *D*, Additional pH profiles of 2 triad mutants (*pink*), along with 14 rSite mutants and the conserved GPR4-H241F mutant (*gray*). Mutants indicated by an asterisk correspond to both rSite and conserved His sites. Black lines in *C* and *D* correspond to wild-type receptors. Data in *B-D* are mean $\pm$ s.e.m. (n = 3-9). Mini G protein V-mGsi was used for all data in panels *C-D* to be consistent with our yeast-based titration data in Fig. 2*D*.

Dataset S1 (separate file). Reagents, buffer, and media

Dataset S2 (separate file). The 430 DCyFIR strains

Dataset S3 (separate file). Yeast and mammalian plasmids

Dataset S4 (separate file). Experimental pH_{50} values
